# Supplementary material for: Investigating the ferric ion binding site of magnetite biomineralisation protein Mms6
Source: PLoS One. 2020 Feb 25;15(2):e0228708. doi: 10.1371/journal.pone.0228708 (PMC7041794; doi:10.1371/journal.pone.0228708)
Supplement: S1 Fig — Predicted structure of Mms6: a) Predicted disordered regions of full length Mms6. Red indicates disorder. b) Mms6 sequence matched to disordered (yellow) and ordered (green) regions within C-terminal region used in SUMO-Mms6 construct. c) Representative simulations of the disordered C- terminal region (yellow) of Mms6. Residue colouring matches that shown in the sequence in b) (DOCX) [file pone.0228708.s001.docx]

**S1. Predicted structure of Mms6:** a) Predicted disordered regions of full length Mms6. Red indicates disorder. b) Mms6 sequence matched to disordered (yellow) and ordered (green) regions within C-terminal region used in SUMO-Mms6 construct. c) Representative simulations of the disordered C- terminal region (yellow) of Mms6. Residue colouring matches that shown in the sequence in b).
