# Supplementary material for: Investigating the ferric ion binding site of magnetite biomineralisation protein Mms6
Source: PLoS One. 2020 Feb 25;15(2):e0228708. doi: 10.1371/journal.pone.0228708 (PMC7041794; doi:10.1371/journal.pone.0228708)
Supplement: S2 Fig — Black boxes indicate codon changes to alanine from the wild-type Mms6 sequence shown. Asterisks highlight residues in Mms6 which were targeted. This figure depicts just the C-terminal region of Mms6 for clarity. The residue numbering corresponds to the residue position in the truncated form of Mms6 isolated by Arakaki et al (1). See cloning section in Supplementary methods section for full sequences of constructs. (DOCX) [file pone.0228708.s002.docx]

**S2.** **Mutagenic primers for the SUMO-Mms6 construct (full length Mms6):** Mutagenic primers used in the experiment. Black boxes indicate codon changes to alanine from the wild-type Mms6 sequence shown. Asterisks highlight residues in Mms6 which were targeted. This figure depicts just the C-terminal region of Mms6 for clarity. The residue numbering corresponds to the residue position in the truncated form of Mms6 isolated by Arakaki *et al* (1). See cloning section in Supplementary methods section for full sequences of constructs.

**
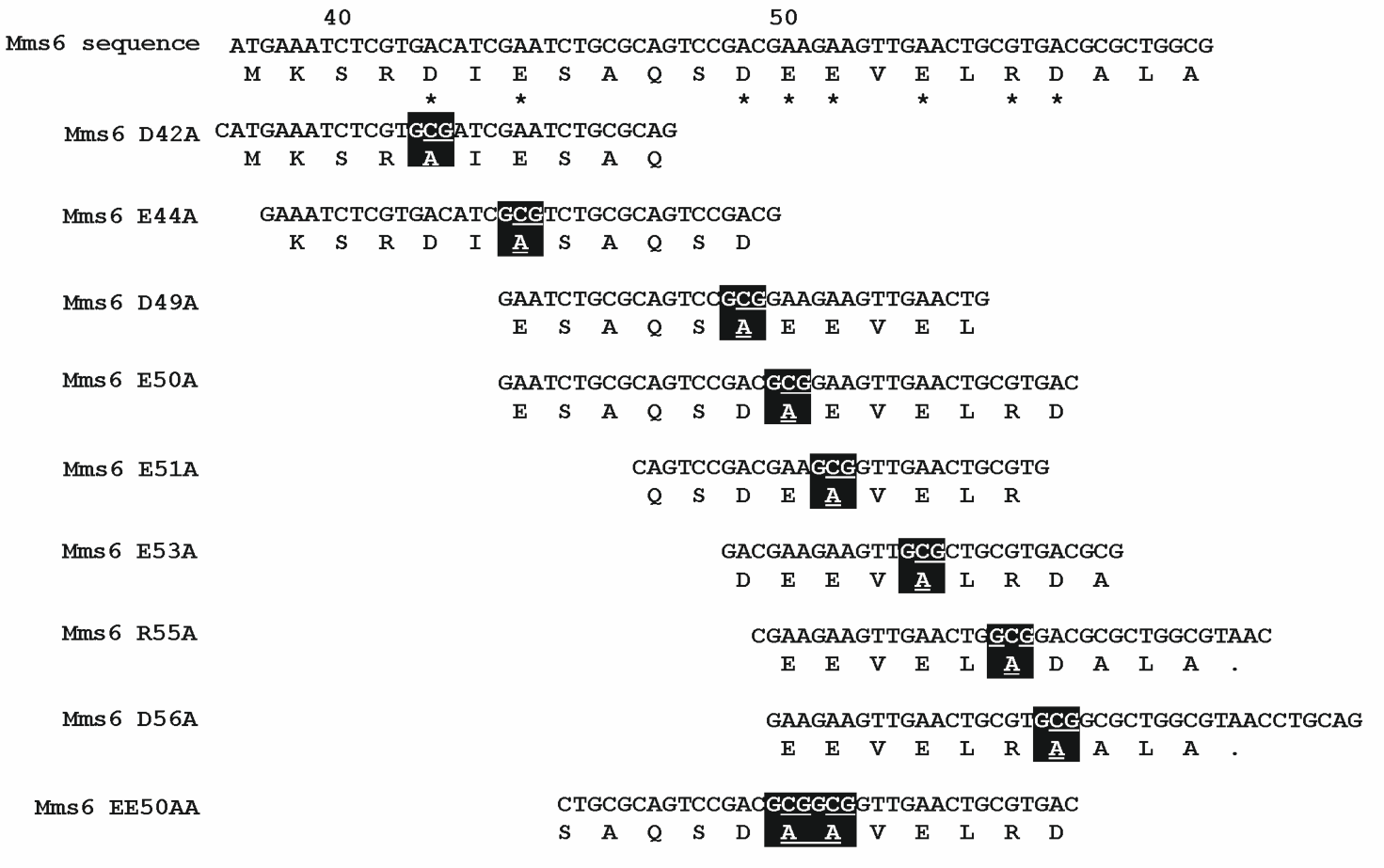
**
