# Supplementary material for: Investigating the ferric ion binding site of magnetite biomineralisation protein Mms6
Source: PLoS One. 2020 Feb 25;15(2):e0228708. doi: 10.1371/journal.pone.0228708 (PMC7041794; doi:10.1371/journal.pone.0228708)
Supplement: S3 Fig — Yield based on UV absorbance at 280 nm and calculated from the theoretical extinction coefficient. This is the yield after purification and dialysis steps were complete. (DOCX) [file pone.0228708.s003.docx]

**S3 Purified Proteins:** Yield of purified SUMO-Mms6 and variants. Yield based on UV absorbance at 280 nm and calculated from the theoretical extinction coefficient. This is the yield after purification and dialysis steps were complete.

| Protein | Yield / mg |
| --- | --- |
| SUMO-Mms6 | 3.81 |
| D42A | 3.28 |
| E44A | 4.21 |
| D49A | 4.99 |
| E50A | 3.17 |
| E51A | 6.48 |
| EE50AA | 2.88 |
| E53A | 7.07 |
| R55A | 1.92 |
| D56A | 6.63 |
