# Supplementary material for: Investigating the ferric ion binding site of magnetite biomineralisation protein Mms6
Source: PLoS One. 2020 Feb 25;15(2):e0228708. doi: 10.1371/journal.pone.0228708 (PMC7041794; doi:10.1371/journal.pone.0228708)
Supplement: S4 Fig — Bio-Rad AnyKd gel (Bio-Rad) with InstantBlue staining (Expedeon, UK). M is the molecular weight marker (PageRuler, Thermo Scientific) with MW in kDa indicated. Lanes 1–10 are: GFP and wildtype SUMO-Mms6, D24A, E44A, D49A, E50A, E51A, EE50AA, E53A, and R55A.Theoretical MW is approximately 21.5 kDa. Apparent monomers and dimers are present in each lane. (DOCX) [file pone.0228708.s004.docx]

**S4 Purified Proteins:** SDS-PAGE analysis of SUMO-Mms6 and variants used in the iron binding study. Bio-Rad AnyKd gel (Bio-Rad) with InstantBlue staining (Expedeon, UK). M is the molecular weight marker (PageRuler, Thermo Scientific) with MW in kDa indicated. Lanes 1-10 are: GFP and wildtype SUMO-Mms6, D24A, E44A, D49A, E50A, E51A, EE50AA, E53A, and R55A.Theoretical MW is approximately 21.5 kDa. Apparent monomers and dimers are present in each lane.

**
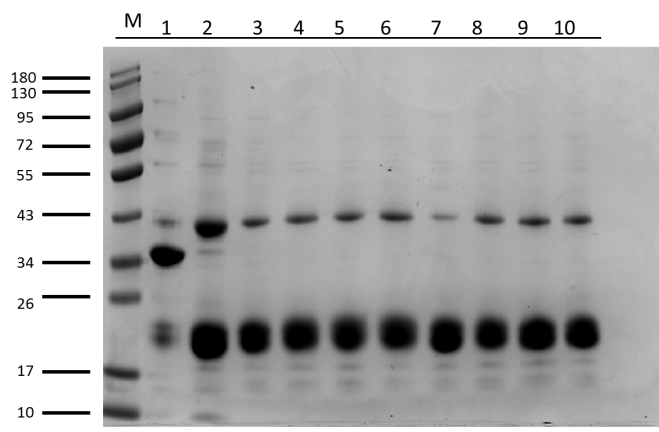
**
