# Supplementary material for: Investigating the ferric ion binding site of magnetite biomineralisation protein Mms6
Source: PLoS One. 2020 Feb 25;15(2):e0228708. doi: 10.1371/journal.pone.0228708 (PMC7041794; doi:10.1371/journal.pone.0228708)
Supplement: S5 Fig — Table shows the calibration standards (Gel Filtration Markers Kit, Merck) and retention volumes from a Superdex 200 10/300 analytical gel filtration column. Gel filtration plot shows the absorbance at 280 nm for a sample of SUMO-Mms6 as it emerges from the same column. Two species (1 & 2) are highlighted, and their calculated molecular weights are presented in the Table below. (DOCX) [file pone.0228708.s005.docx]

**S5 Purified Proteins:** Gel filtration analysis of SUMO-Mms6. Table shows the calibration standards (Gel Filtration Markers Kit, Merck) and retention volumes from a Superdex 200 10/300 analytical gel filtration column. Gel filtration plot shows the absorbance at 280 nm for a sample of SUMO-Mms6 as it emerges from the same column. Two species (1 & 2) are highlighted, and their calculated molecular weights are presented in the Table below.


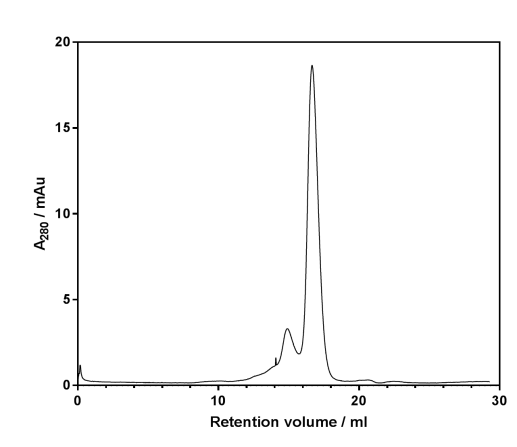


Species 1

Species 2

| Protein/Calibration standard | MW / kDa | Retention volume / ml |
| --- | --- | --- |
| Blue dextran | - * | 8.1 |
| Beta amylase | 200 | 11.66 |
| Alcohol dehydrogenase | 150 | 12.59 |
| Albumin | 66 | 13.96 |
| Carbonic anhydrase | 29 | 16.28 |
| Cytochrome C | 12.4 | 17.8 |
| Species 1 | 22.5** | 16.60 |
| Species 2 | 46.1** | 15.03 |

*Emerges at column void volume. ** Calculated from calibration standards.
