# Supplementary material for: Investigating the ferric ion binding site of magnetite biomineralisation protein Mms6
Source: PLoS One. 2020 Feb 25;15(2):e0228708. doi: 10.1371/journal.pone.0228708 (PMC7041794; doi:10.1371/journal.pone.0228708)

**S6 Purified Proteins:** SDS-PAGE analysis of Mms6 and Mms6MM. BisTris RunBlue gel with Instant Blue staining (Expedeon, UK). M is the molecular weight marker (PageRuler, Thermo Scientific) with MW in kDa indicated. Purified Mms6MM and Mms6 are shown.


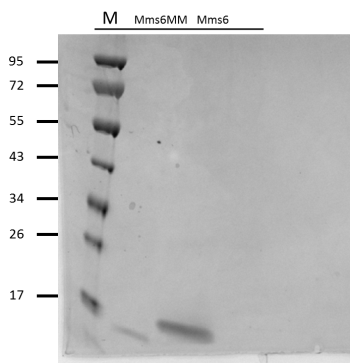

Supplement: S6 Fig — BisTris RunBlue gel with Instant Blue staining (Expedeon, UK). M is the molecular weight marker (PageRuler, Thermo Scientific) with MW in kDa indicated. Purified Mms6MM and Mms6 are shown. (DOCX) [file pone.0228708.s006.docx]
