# Supplementary material for: Investigating the ferric ion binding site of magnetite biomineralisation protein Mms6
Source: PLoS One. 2020 Feb 25;15(2):e0228708. doi: 10.1371/journal.pone.0228708 (PMC7041794; doi:10.1371/journal.pone.0228708)
Supplement: S7 Fig — Scaling maps for IDRs in Mms6 and mutants. Cooler colours reflect inter-residue compaction compared to the behavior of an excluded volume model, while warmer colours reflect expansion. (DOCX) [file pone.0228708.s007.docx]

**S7. Computational Analysis:** Scaling maps for IDRs in Mms6 and mutants. Cooler colours reflect inter-residue compaction compared to the behavior of an excluded volume model, while warmer colours reflect expansion.

**
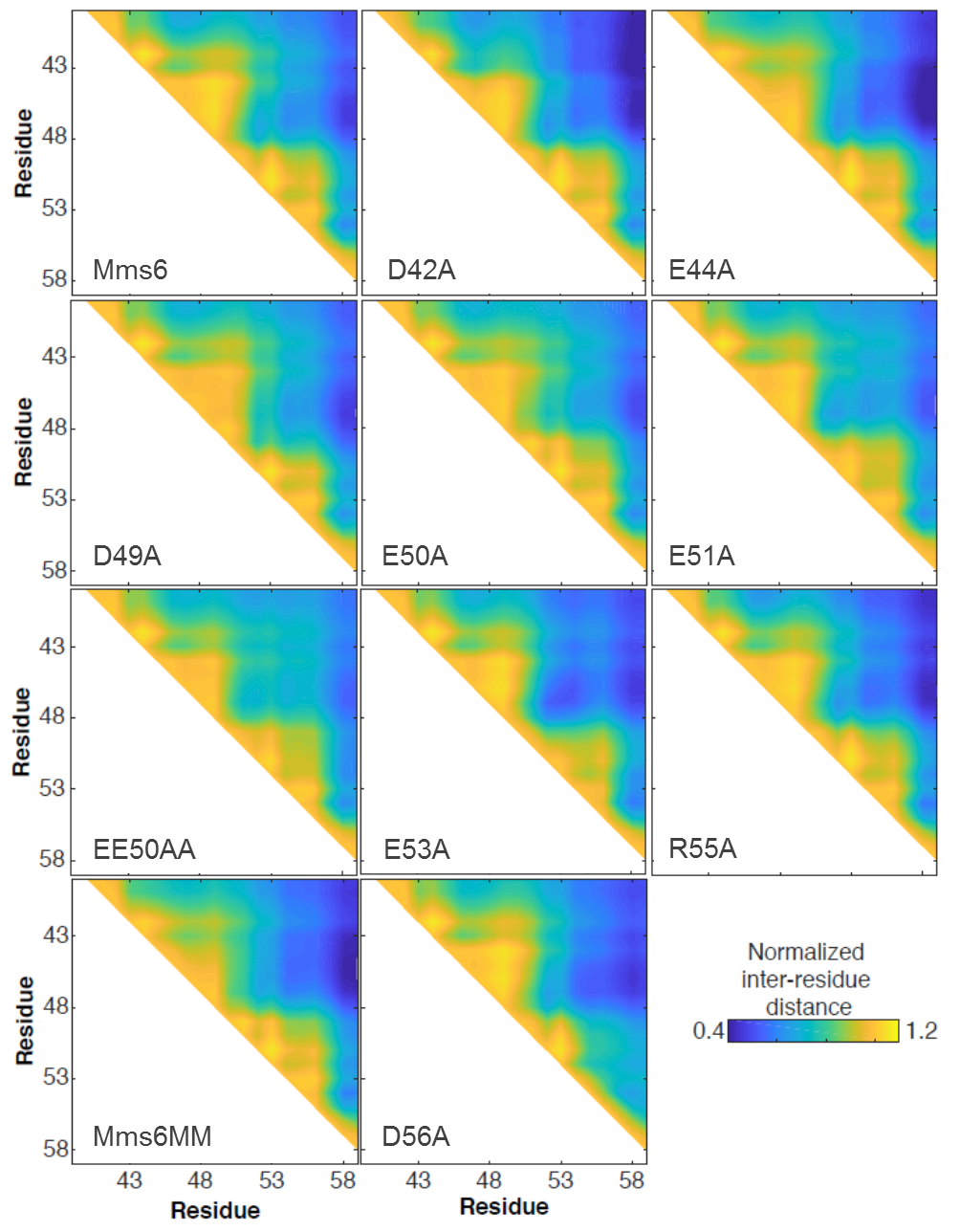
**
