# Supplementary material for: Investigating the ferric ion binding site of magnetite biomineralisation protein Mms6
Source: PLoS One. 2020 Feb 25;15(2):e0228708. doi: 10.1371/journal.pone.0228708 (PMC7041794; doi:10.1371/journal.pone.0228708)
Supplement: S8 Fig — Colours are a probability range from blue (zero probability) to yellow (0.004 probability). (DOCX) [file pone.0228708.s008.docx]

**S8. Computational Analysis:** Conformational distributions from atomistic simulations of the Mms6 and mutants in terms of size and shape (asphericity). Colours are a probability range from blue (zero probability) to yellow (0.004 probability).

**
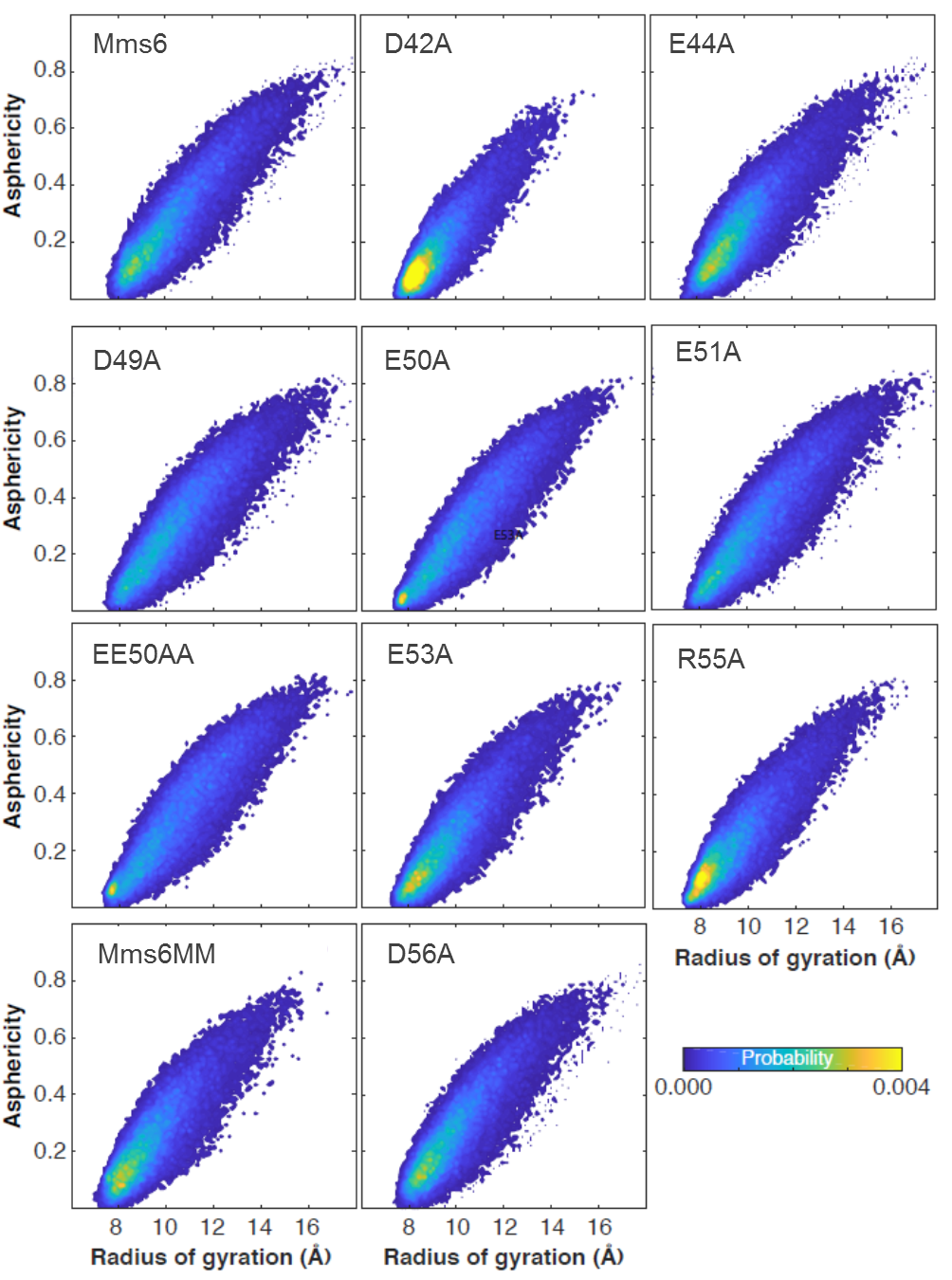
**
